# Proteomic Profiling of GLP-1-Mediated Cardioprotection in a Large Animal Model of Chronic Coronary Artery Disease

**DOI:** 10.18103/mra.v13i11.7114

**Published:** 2025-11

**Authors:** Clark Zheng, Christopher Stone, Kelsey Muir, Dwight Harris, Frank W. Sellke

**Affiliations:** 1 Division of Cardiothoracic Surgery, Department of Surgery, The Warren Alpert Medical School of Brown University

**Keywords:** Coronary Artery Disease, Large Animal Model, Translational Research, GLP-1 Agonist, Semaglutide, Cardiac Metabolism, Proteomics

## Abstract

**Background:**

Coronary artery disease (CAD) imposes marked morbidity on patients, with many afflicted with debilitating residual symptoms despite optimal application of the available medical and surgical options. Glucagon-like peptide-1 (GLP-1) agonists have emerged from the resultant search for adjuncts as promising cardioprotective candidates in clinical trials.

**Aims:**

We have previously characterized the augmented myocardial functional response to GLP-1 agonism; in this experiment, we aim to elucidate the molecular basis of this augmentation using highly sensitive proteomic analysis.

**Methods:**

Yorkshire swine underwent surgical induction of CAD-associated ischemic cardiomyopathy through ameroid constrictor placement. Postoperatively, all were allocated either to receive semaglutide (n=6), or no drug (n=10) for 5 weeks, whereupon animals underwent myocardial resection and sectioning. The most ischemic ventricular sections were identified, from which tissue aliquots were fractionated using high-performance liquid chromatography and analyzed using mass spectrometry.

**Results:**

There were 594 upregulated and 90 downregulated proteins identified in the semaglutide cohort compared with control cohort. Enrichment analysis revealed marked upregulation of multiple central metabolic pathways, including the glycolytic and tricarboxylic acid cycle pathways. The significantly downregulated proteomic fraction was found within pathways relevant to the induction of dilated and hypertrophic cardiomyopathy.

**Conclusions:**

Myocardial sections taken from semaglutide-treated animals exhibited a striking and multifaceted increase in metabolic flexibility. This result implicates enhanced resilience against the energetic strain imposed by ischemic disease as a mechanistic account of GLP-1-mediated cardioprotection.

## Introduction:

Despite the progress that has been made in recent years, coronary artery disease (CAD) remains the leading cause of death, both in the United States and worldwide.^1,2^ Moreover, mortality alone is an insufficient means to measure the impact of CAD, as it is also the leading global cause of cardiovascular morbidity, imposing over 200 million disability-adjusted life years on patients living with the sequelae of ischemic cardiomyopathy.^3^ These data provide the rationale for extensive performance of surgical and percutaneous revascularization procedures in patients with CAD: over 600,000 such procedures are performed yearly in the United States across a population of 20 million patients with established disease.^4^

Unfortunately, while this procedural volume is supported by the extent of disease it aims to address, it is far from a perfect solution. Even in contemporary series, as many as 33% of surgically and over 50% of percutaneously revascularized patients are left with residual lesions post-procedurally.^5,6^ Risk is still graver for an additional cohort with CAD determined to be refractory to all available treatments, with numbers estimated to reach 1.8 million in prevalence and an additional 50,000 in annual incidence in the United States.^7^ Therefore, there is a substantial population of patients, numbering in the millions worldwide, living with disabling cardiogenic pain without hope of relief. This refractory fraction of CAD, especially given its procedure-resistant microvascular nature, requires urgent development of pharmacological adjuncts to the current armamentarium.^8^ Recognition of this has spurred a number of innovative approaches that, despite their preclinical promise, have yielded disappointing efforts at translation into a meaningful clinical role.^9,10^ The cause of this translational failure is largely due to the use of imprecise models, and its cumulative effect is substantial.^11^

One way to circumvent these challenges is exemplified by the therapeutic trajectory of glucagon-like peptide-1 (GLP-1) agonists. Characterized initially for their role in postprandial stimulation of pancreatic insulin secretion, these agents found original clinical use in the early 2000s for the reduction of hyperglycemia.^12^ Studies conducted to evaluate for cardiovascular safety thereafter resulted in a vast expansion of applications. This was driven especially by the reduced major adverse cardiovascular events found to result from the addition of these drugs to antidiabetic regimens.^13^ Accordingly, approved indications expanded in early 2024 to include not only reduction of hyperglycemia, but also weight management in non-diabetic patients, and even cardiovascular risk reduction in patients with elevated weight and comorbid cardiovascular disease.^14^

The properties of GLP-1 agonists render them suited not only for reduction in cardiovascular risk, but also a potentially broad array of associated uses. Attempts at this have already been initiated, through the preclinical and clinical studies that have been conducted for conditions as diverse as hypertension, heart failure, and coronary artery disease. The results of these experiments have been favorable, with benefits speculated to arise from metabolic modulation, cardiomyocyte apoptotic inhibition, reduced cardiac inflammation, and augmented endothelial function.^15^ This body of evidence, given its generation largely in small animal- and cell culture-based models, is suggestive, but not sufficient. This point is effectively made in a recently conducted large trial, which states that, “our understanding of the mechanisms of cardiovascular protection with [the GLP-1 agonist] semaglutide remains speculative…clinical benefit is [thought to be] achieved through multiple interrelated pathways.”^16^

Acknowledgement of this evidence gap prompted our lab, through use of a large animal model of CAD that replicates the features of disease as it occurs in humans,^17^ to investigate the cardiac consequences of semaglutide administration. Our model has been deliberately isolated from the influence of the metabolic comorbidities that currently frame the use of this drug, with the goal of revealing any direct effects GLP-1 agonists may exert on cardiac tissue. We have previously characterized several notable benefits through this work, including improved left ventricular performance, increased coronary perfusion to the ischemic myocardial region, diminished apoptotic death of ischemic myocytes, and reduced interstitial and perivascular fibrosis within the perfusion-restricted territory.^18^ With this study, we set out to explore the molecular underpinnings of these physiologic changes using proteomic profiling. Our overall aim was to contribute mechanistic specificity to the task of selecting patients most likely to benefit from GLP-1 agonist therapy.

## Methods:

### ANIMAL MODEL AND ASSOCIATED OVERSIGHT:

**Figure 1.**
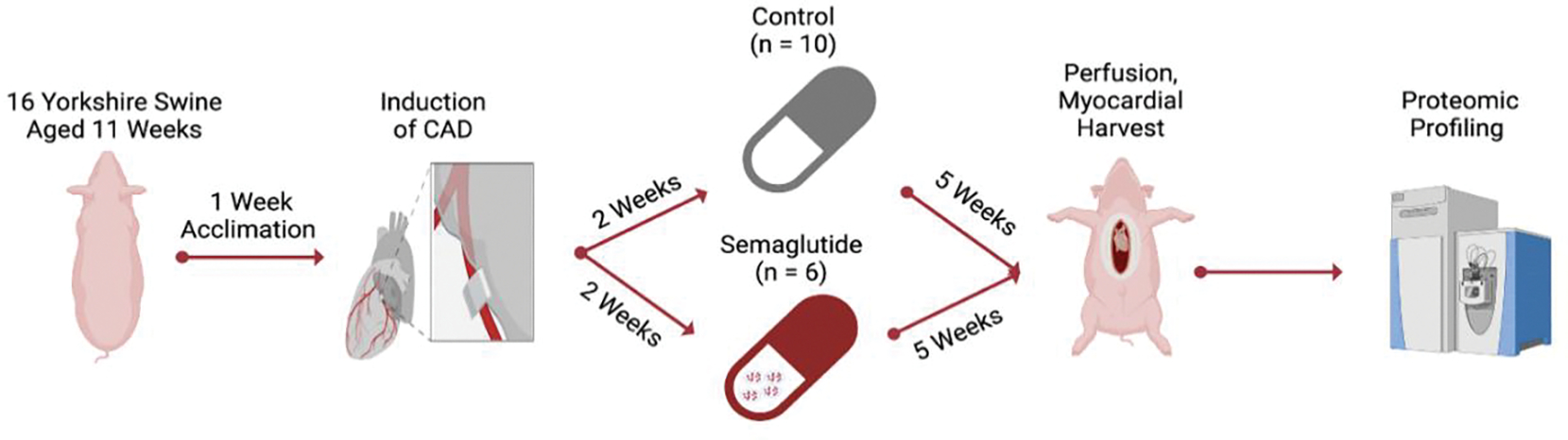
Coronary Artery Disease Induction, Tissue Harvest, and Proteomic Analysis Protocol Flowchart. Beginning with the one-week interval between arriving at the veterinary facility and the initiating surgery, all steps that occur at a fixed point along the experimental timeline are designated over the red arrows between steps. CAD: coronary artery disease.

All steps of our protocol were designed and carried out under supervision by the Institutional Animal Care and Use Committee at Rhode Island Hospital and veterinary staff employed by Brown University (protocol #505821). The protocol, depicted in Figure 1, began with the arrival of animals (Yorkshire swine, n=16, of which 7 were female and 9 were intact males), to our facility from the Cumming School of Veterinary Medicine, Tufts University, Grafton, MA. Animals were 10 weeks of age on arrival, and were fed a standard laboratory swine diet, formulated for balanced nutrition and growth (#8753, composed of 16% protein, 33% carbohydrates, and 3.9% fat for a total of 2.3 kcal/g, Envigo, Indianapolis, IA) during a one-week acclimation period. After acclimation, all animals are transitioned to the surgical stage of the protocol, which begins with the placement of an ameroid constrictor device designed to induce chronic CAD.

### AMEROID CONSTRICTOR PLACEMENT:

Presurgical preparation began with initiation of antithrombotic therapy (aspirin, 10 mg/kg). This was continued for 5 days postoperatively due to data suggesting that this procedure produces optimal ischemic territory size consistency between animals.^19^ Surgeons were unaware of animal treatment group allocation at the time of procedure performance, and therefore lacked any inducement to conduct cases non-homogenously. Anesthesia was initiated intramuscularly (4.4 mg/kg telazol and 2.2 mg/kg xylazine) and subsequently maintained with an inhaled halogenated anesthetic (isoflurane, 0.75–3.0%). Following a standard iodinated antimicrobial prep and intramuscular antibiotic injection (30 mg/kg cephalexin), swine were positioned on a rightward lateral tilt from supine for optimal myocardial orientation.

After injection of anesthetics along the anticipated incisional track (5 mL, 2% lidocaine), an anterolateral thoracotomy was carried out within the left third thoracic interspace. The pericardium overlying the left atrial appendage was exposed and entered sharply. A Satinsky-Debakey vascular clamp was used to atraumatically grasp the superior ridge of the left atrial appendage, a corner of which was encircled with a silk tie and used to retract the appendage. This exposed the proximal course of the left circumflex coronary artery (LCx) just distal to its bifurcation from the left main coronary artery. Once a satisfactory length of LCx was exposed, the animal was heparinized (80 IU/kg), and a silastic vessel loop was passed to encircle the vessel. This was positioned prior to the takeoff of the obtuse marginal branch of the LCx to create consistency across animals in ischemic territory generation and gently retracted for 2 minutes. The electrocardiographic tracing was monitored during this time for ST segment changes to ensure effective focal ischemic induction. With ischemia confirmed, gold microspheres (5 mL, containing approximately 7.5 million total spheres, BioPAL, Worcester, MA) were injected into the left atrial appendage. Because the myocardial tissue supplied by the LCx is occluded during injection, these microspheres distribute to all cardiac capillary beds apart from those in the left lateral free wall, allowing precise delineation of the ischemic territory.

When injection is completed, the ameroid constrictor device (Research Instruments SW, Lebanon, OR) is placed around the vessel at the location of vessel loop occlusion. Closure of the pericardium, rib space, subcutaneous tissue, and skin sequentially then completes the case. Animals are recovered with initiation of a multimodal analgesic protocol supplemented as needed to prevent distress (fentanyl patch and buprenorphine, 4 μg/kg and 0.03 mg/kg, respectively).

### ORAL SEMAGLUTIDE TREATMENT:

The ameroid constrictor is composed of two rings, concentrically arrayed and divided by a keyhole that admits the vessel. The outer, rigid ring is titanium, while the inner is made of hygroscopic, compressed casein that swells as it absorbs pericardial fluid over the course of several weeks, ending in total occlusion^20^. While this occlusion and the corresponding ischemic territory is generated focally and in this respect differs from the more diffuse atherosclerotic disease seen in patients, it is performed in this model to maximize model homogeneity between groups. Animal numbers were arbitrarily assigned to treatment groups following ameroid constrictor surgery survival, at which point investigators possessed no knowledge of their cardiac or broader health status. In animals randomized to receive treatment, oral semaglutide (Novo Nordisk, Bagsværd, Denmark, initially 1.5 mg daily and scaled up at two weeks to 3 mg) was begun on the 3^rd^ postoperative week and continued for 5 full weeks. The chosen dose was proportional by weight to that used in a cardiovascular outcomes trial, but scaled to the weight of experimental animals, which was approximately half that used in patients enrolled in the cardiovascular outcomes trial conducted using oral semaglutide; these patients initially received 3 mg oral semaglutide daily, and were-subsequently up-titrated to 7 mg ^21^. Initiating treatment after ameroid closure has proceeded for several weeks replicates the clinical context of drug initiation in patients already diagnosed with CAD.^22^ Treatment allocation generated two total animal groups: semaglutide-treated (SEM, n=6), and control counterparts that received no drug (CON, n=10). All animals were maintained under identical conditions postoperatively, including the provision of a standard porcine chow diet (Teklad Miniswine Diet 8753, Indianapolis, IA).

### CARDIAC FUNCTIONAL ANALYTICS, RESECTION, AND SECTIONING

After initiation of analgesia, anesthesia, and aseptic preparation as in the previous procedure, swine were placed supine to undergo median sternotomy. Pericardial division and intrapericardial lysis of adhesions was performed as needed to expose the heart and great vessels. Following acquisition of functional and perfusion-related measurements as previously described,^18^ all recording instruments were removed, and the procedure was concluded by division of the heart from its thoracic and vascular attachments. The resected heart was then delivered to an adjacent table for anatomical sectioning of the left ventricular myocardial tissue, first into apical and basal halves, and then radially according to the course of the major coronary arteries. The resultant sections were then segmented further, with one component designated for drying and neutron activation for determination of microsphere counts, and another for flash freezing in liquid nitrogen for proteomic profiling.^23^ Because the LCx was occluded during gold microsphere injection, the myocardial segment supplied uniquely by the proximal LCx displayed the lowest gold microsphere count. This segment represented the most ischemic myocardial component. Given that the purpose of this study was to explicate the direct effects of semaglutide administration on the ischemic myocardium, the most ischemic sections served as the source for proteomic analysis. Notably, microsphere quantification is performed by technicians from an outside laboratory unaware of experimental circumstances. Accordingly, the tissue that is determined as ischemic and therefore studied is differentiated in a manner to which experimenters were blinded. An additional element of blinding arises from the use of independent technicians to perform proteomic experiments. The result of this is that tissue generation, tissue processing, and tissue analysis were all performed in a blinded, independent fashion.

### PROTEOMICS

Proteomics was completed through collaboration with a dedicated core at the University of Massachusetts-Boston that was unaware of experimental details and uninvolved with tissue harvesting or selection. The process began with delivery of deep-frozen ischemic left ventricular to the core. All segments were thawed, weighed, and then pulverized for resuspension in T-PER with Halt Protease Inhibitor and PhosSTOP (the former two from Thermo Fisher Scientific, Waltham, MA; the latter from Sigma-Aldrich, St. Louis, MO). A bicinchoninic assay was performed to determine protein concentration, on the basis of which 100 μg aliquots were buffer-exchanged by addition of chilled acetone for overnight precipitation at −20°C. Protein pellets were extracted from these aliquots by two rounds of centrifugation at 16,000 rpm × g, first for 10 minutes, and then for 5 minutes after washing with 50 μL of chilled acetone. Ammonium bicarbonate was used to resuspend these pellets. Following alkylation with 500 mM iodoacetamide and reduction with 500 mM DTT, pellets were digested overnight at 37°C with 1 mg/mL Trypsin/Lys-C.

Using 1 μL from each digested sample, high-performance liquid chromatography (HPLC) was performed using the UltiMate 3000 apparatus (Thermo Fisher, San Jose, CA). The system was set to a flow rate of 0.060 mL/min and composed of 5 mM ammonium formate in a gradient of water/acetonitrile concentrations (all mobile phase solvents were Optima LC/MS Grade, and purchased from Fisher Scientific, Fair Lawn, NJ). An Acquity ultra-performance liquid chromatography BEH C18 column (Waters, Milford, MA) was used to separate the samples, which were thereby readied for analysis using liquid chromatography-ion mobility-mass spectrometry. This was performed using a coupled online system consisting of both the Bruker trapped ion mobility time of flight mass spectrometer (Bruker Scientific, Billerica, MA), and the Evosep One liquid chromatography platform (Evosep, Odense, Denmark). This spectrometry system exhibits superior capacity for reproducible peptide identification and quantification of biological samples when compared with a precursor system.^24^

Samples were first processed by data-dependent acquisition for generation of a spectral library, for which spectra were streamed from the spectrometer platform directly into a workstation and searched against both known contaminants and against a porcine (*Sus scrofa*, downloaded on August 22, 2023) protein database. Fragment ion tolerance was set to 30 ppm and precursor tolerance to 20 ppm, with one tryptic terminus per peptide and one peptide per protein established as prerequisites identification, and with up to two variable modifications per peptide permitted. The yield of these parameters was then assembled, filtered, and validated with DTASelect software, using a false discovery rate (FDR) of 0.01.^25^ All samples were then processed again using data-independent acquisition, in this case using 20 ppm and 15 ppm for precursor and fragment ion tolerance, respectively, against the prefabricated spectral library of 69,797 precursors to maximize discovery, precision, and reproducibility of the proteomic spectrum.

### PROTEOMIC STATISTICAL ANALYSIS

**Figure 2.**
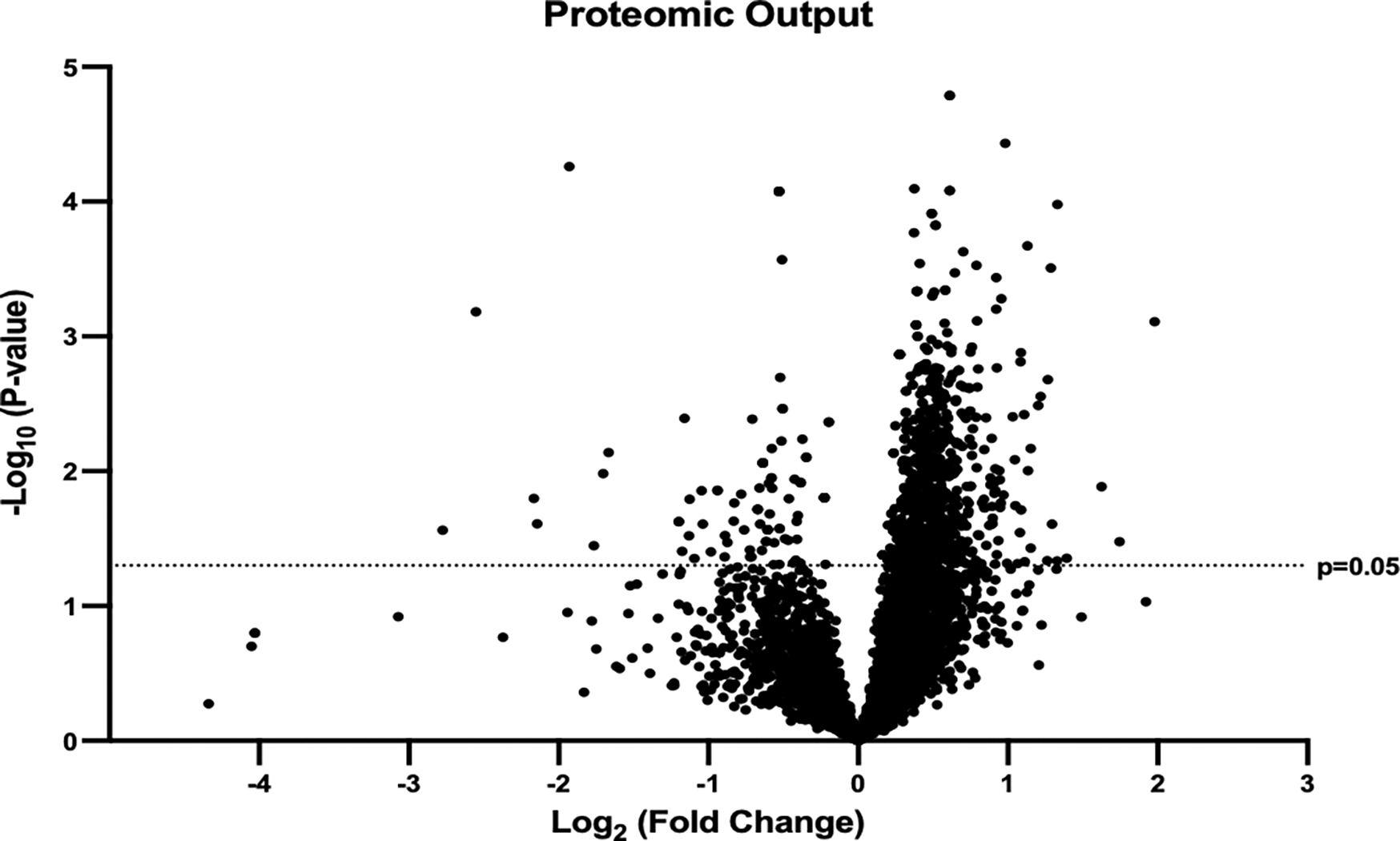
Proteomic Volcano Plot. A total of 5588 differentially expressed proteins were identified. A line intersects the y-axis at the point corresponding to p=0.05. The x-axis represents the log_10_ of all p-values, while the y-axis displays the fold change of mean protein expression as log_2_ values.

The output of this sequence was a large dataset that, prior to filtration, identified expression intensities of 7,322 proteins in common between SEM and CON groups. Although these data were subjected to match between runs processing and globally normalized, they were also subjected to three additional filtration steps prior to processing. This was done in an effort to assess only peptides most likely to constitute components of true biological changes attributable to treatment. First, all proteins insufficiently abundant to meet the identification threshold in greater than 50% of samples within either group were discarded. This resulted in a reduction of the total number of proteins subjected to analysis to 5588; see Figure 2 for a graphical display of the product as a volcano plot. Then, in keeping with conventions in the field,^26^ all mean abundance differences between groups of p<0.05 using two-tailed t testing and fold changes below 1.1 were also discarded. Moreover, as only abundance data for which both SEM and CON groups exhibited normal distributions is appropriate for parametric analysis, Shapiro-Wilk testing was used to remove all proteins for which data distributions were non-normal in either group. Finally, cleaned data underwent graphical enrichment using the ShinyGO (gene ontology, version 0.77) to produce the pathway assessment reported below.^27^

## Results:

### SEMAGLUTIDE PRODUCES PROFOUND ISCHEMIC MYOCARDIAL METABOLIC MODULATION

**Figure 3.**
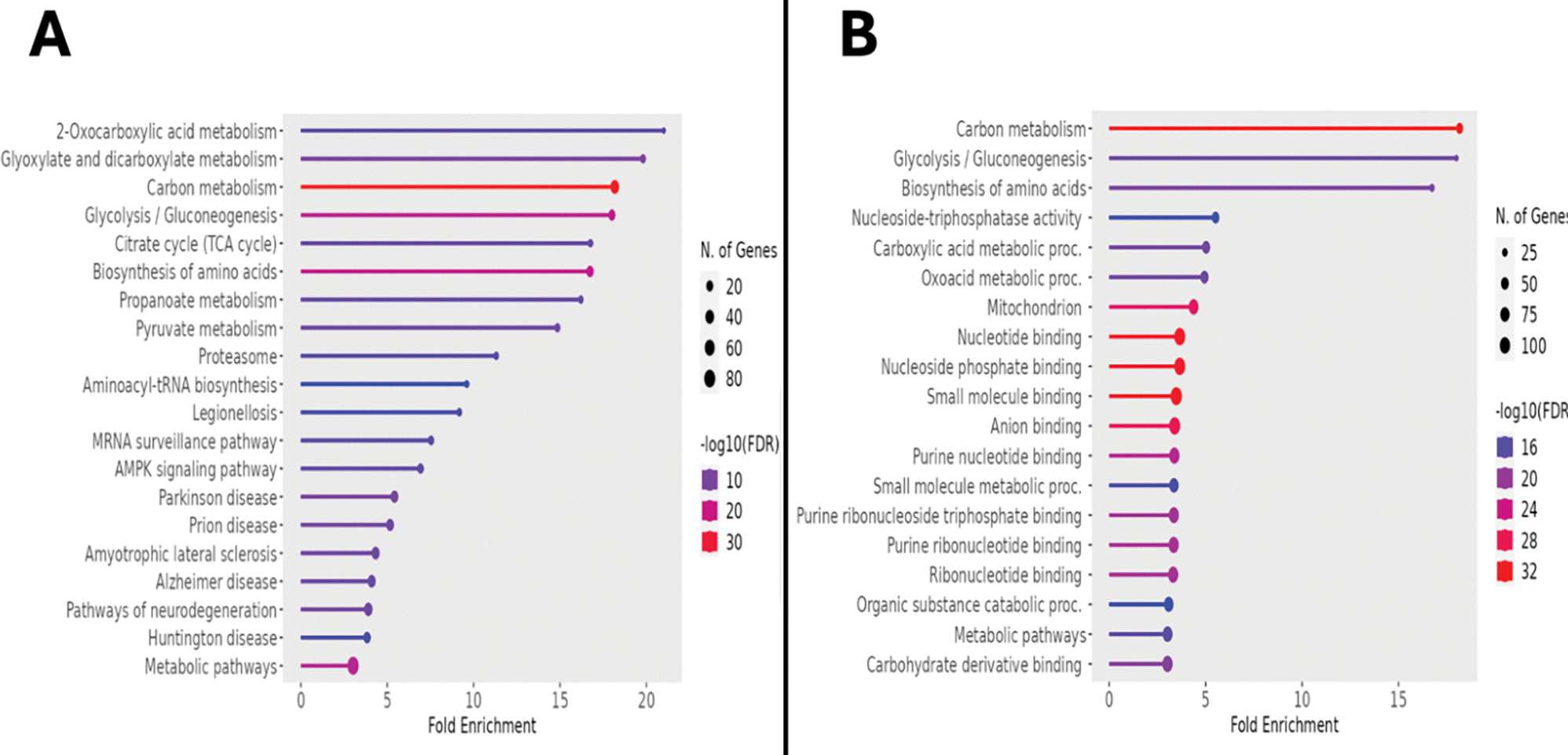
Semaglutide Exerts a Widespread Metabolic Modulatory Effect. **3A:** Lollipop plot showing the KEGG-based interpolation of upregulated proteins in treated animals and corresponding enriched metabolic pathways. The diameter of the terminal circle indicates number of genes, colors represent the log_10_ (FDR), and x-axis indicates fold change. **3B:** Lollipop plot of the same dataset interpolated into all available databases of ShinyGO 0.77 revealed similar findings. FDR: false discovery rate.

From the initial output of over 7,000 proteins, restriction for statistical significance, fold change, and abundance identified 594 upregulated proteins. Transfer of the associated accession numbers into the Kyoto Encyclopedia of Genes and Genomes (KEGG) for bioinformatic analysis demonstrated a multitude of metabolic alterations, as indicated by the fold enrichment of associated pathways represented within the dataset. These pathways included 2-oxocarboxylic acid metabolism, dicarboxylate metabolism, glycolysis/gluconeogenesis, the tricarboxylic acid cycle, and AMPK signaling, as displayed in Figure 3A. Table 1 presents these data, including the false discovery rate (FDR), which, analogous to a p-value, represents the likelihood that the associated enrichment is due to chance; a cutoff of 0.05 was used when generating this data, but note that all FDR values are far below this. Generalizing from KEGG to all available databases for ontologic corroboration produced similar results, as depicted in Figure 3B. There was marked enrichment according to this analysis in glycolysis/gluconeogenesis, amino acid biosynthesis, carboxylic acid metabolism, and oxoacid metabolism.

**Figure 4.**
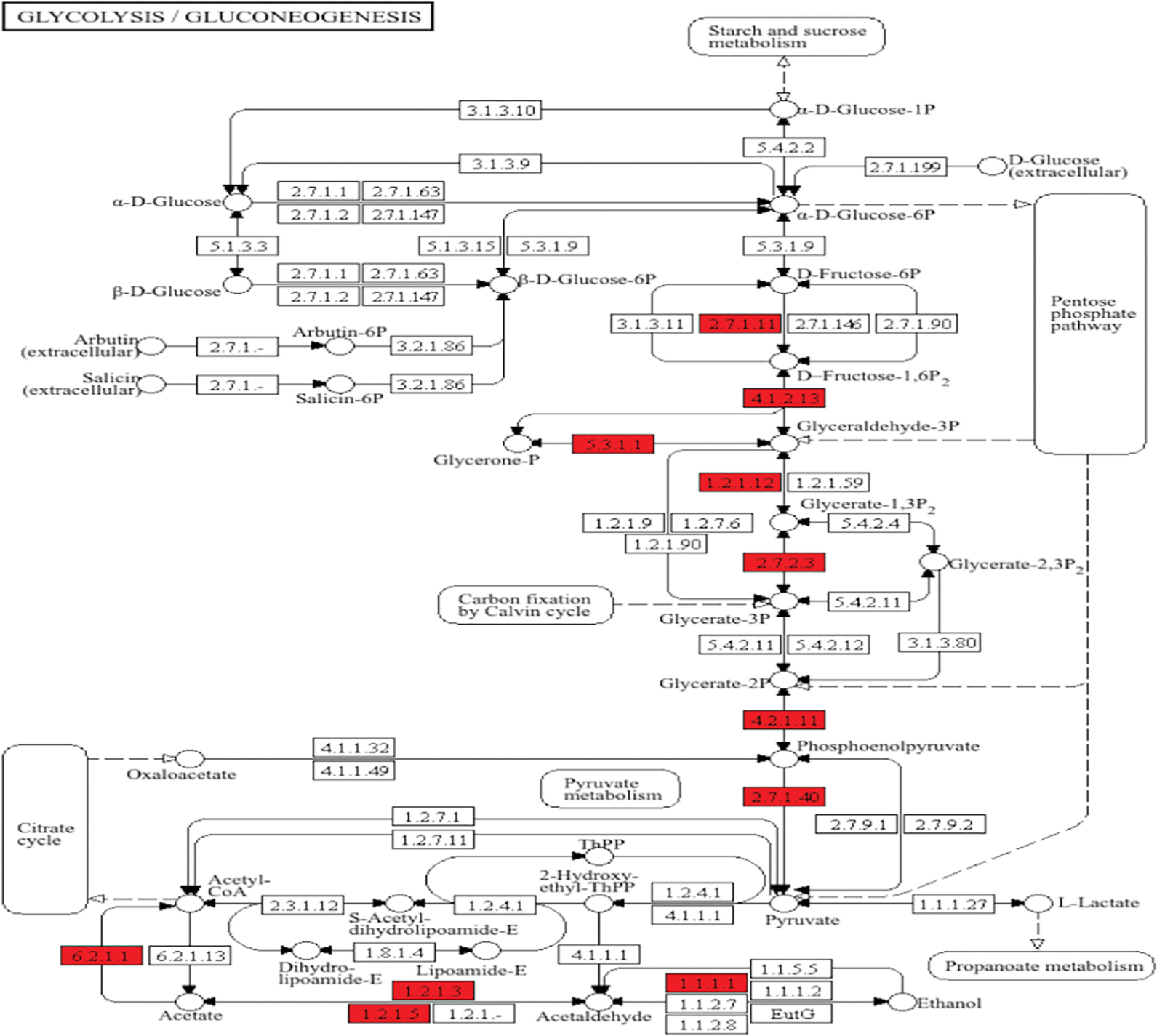
Semaglutide Increases Glycolysis Pathway Enzyme Expression. This schematic depicts the cytosolic catabolism of glucose for cellular energy generation. Enzymes found to be significantly upregulated in treated animals are highlighted in red. 2.7.1.1: phosphofructokinase; 4.1.2.13: aldolase; 5.3.1.1: triose-phosphate isomerase; 1.2.1.12: glyceraldehyde-3-phosphate dehydrogenase; 2.7.2.3: phosphoglycerate kinase; 4.2.1.11: enolase, 2.7.1.40: pyruvate kinase; 1.1.1.1: NAD-dependent alcohol dehydrogenase; 1.2.1.3: NAD-dependent aldehyde dehydrogenase; 6.2.1.1: acetyl-CoA synthase.

**Figure 5.**
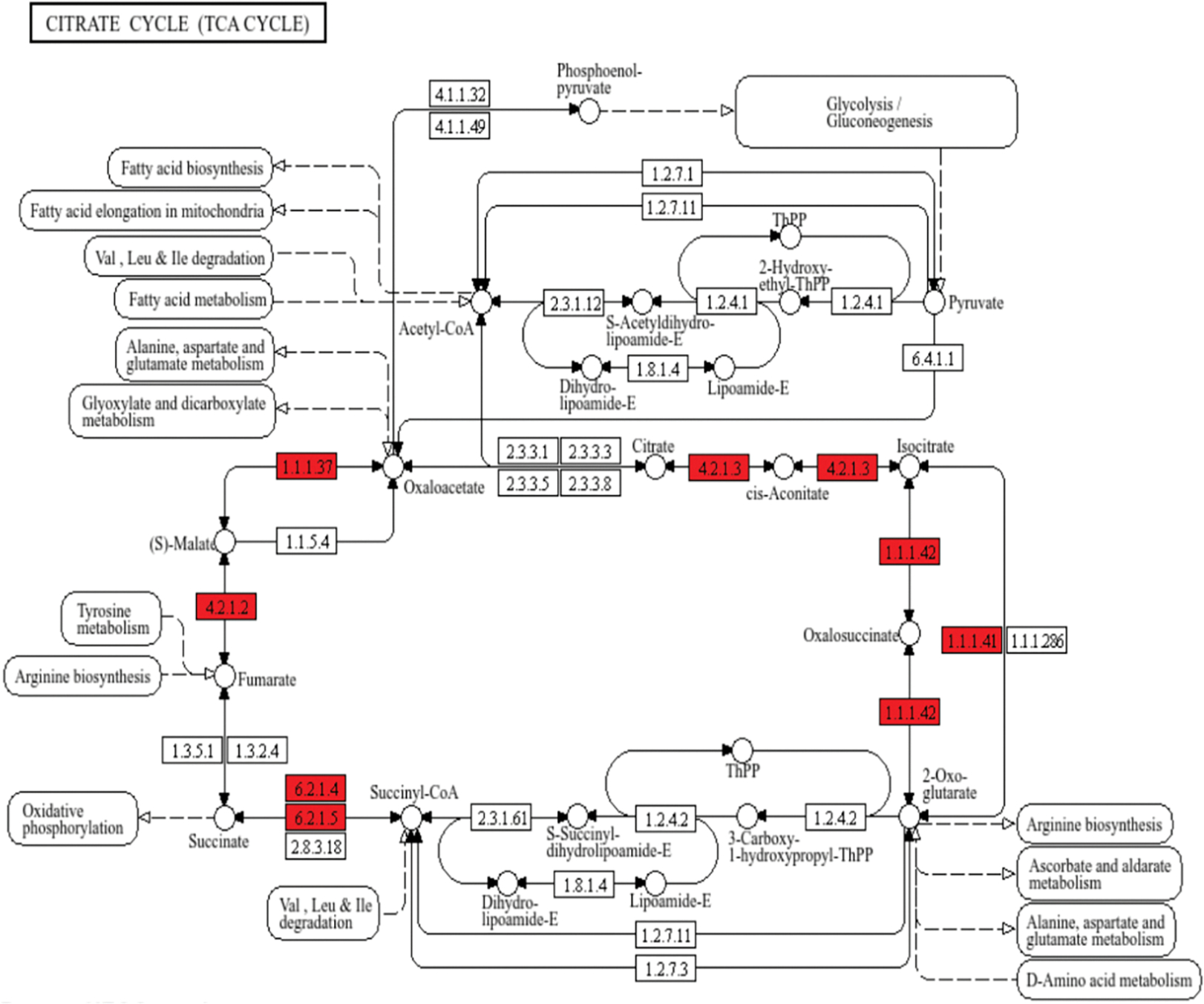
Semaglutide Activates TCA Cycle-Mediated Metabolism. Semaglutide treatment was associated with upregulations in nearly all enzymes critical to TCA cycle flux. Upregulated enzymes are highlighted in red. 4.2.1.3: aconitase; 1.1.1.41/42: isocitrate dehydrogenase; 6.2.1.4/5: succinyl-CoA synthetase; 4.2.1.2: fumarase; 1.1.1.37: malate dehydrogenase.

To further describe this bioinformatic output, KEGG-based pathway analysis was performed, in which upregulated proteins were positioned within the functional processes of porcine metabolism.^28^ Of the approximately 90 metabolic enzyme networks available for such analysis, glycolysis/gluconeogenesis and the tricarboxylic acid (TCA) cycle were chosen, given both their relevance to normal myocardial metabolism, and their inclusion among the list of the most upregulated processes in the semaglutide-treated ischemic myocardial proteome.^29^ As shown in Figure 4, there are 11 enzymes relevant to glycolysis/gluconeogenesis upregulated in this dataset, including phosphofructokinase, aldolase, glyceraldehyde-3-phosphate dehydrogenase, phosphoglycerate kinase, enolase, and pyruvate kinase. Parenthetical numbers indicate the Enzyme Commission designations corresponding to upregulated enzymes in the diagram. Of these, upregulations in phosphofructokinase and pyruvate kinase are of particular note, as these represent rate-limiting catalytic steps within the glycolytic pathway, which is a described result of GLP-1 receptor stimulation that ultimately results in augmented ATP production.^30^ Figure 5 shows results for the citric acid cycle, in which 8 enzymes were upregulated within this dataset, including aconitase, the rate-limiting isocitrate dehydrogenase, succinyl CoA synthetase, fumarase, and malate dehydrogenase.

### SEMAGLUTIDE REDUCES RESPIRATORY AND CARDIOMYOPATHIC PATHWAY EXPRESSION

**Figure 6.**
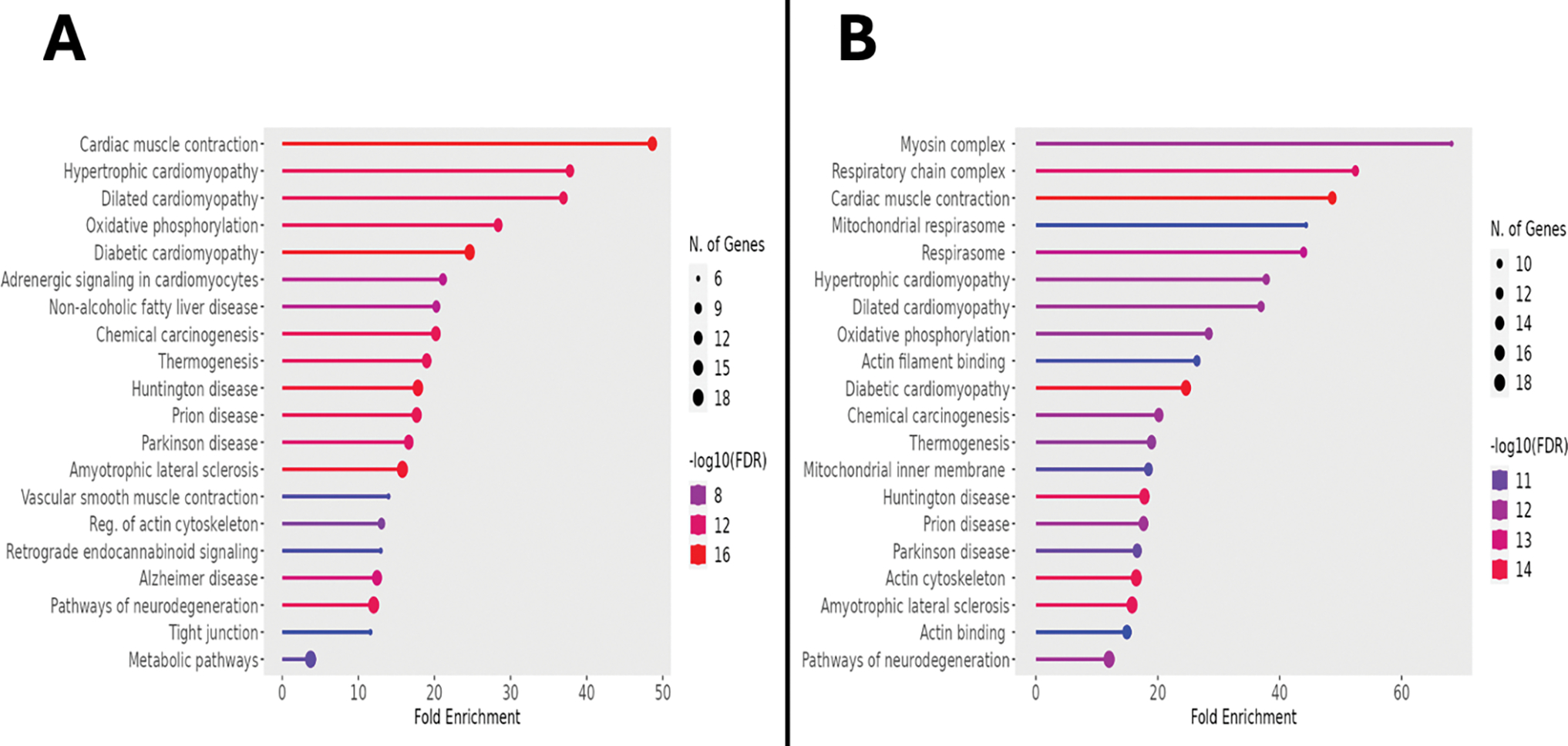
Semaglutide Reduces Cardiomyopathic Pathway and Respiratory Complex Subunit Expression. **6A:** Lollipop plot displaying the KEGG-generated bioinformatic yield derived from the downregulated proteome of treated ischemic myocardial tissue. The diameter of the terminal circle indicates number of genes, colors represent the log_10_ (FDR), and x-axis indicates fold change. **6B:** When processed using all available databases, the same inputs yielded a similar set of pathways. FDR: false discovery rate.

Using the same thresholding as described above for upregulated proteins, there were 90 downregulated proteins in the semaglutide-treated animals. When processed in KEGG for bioinformatic surveillance, this dataset provided additional evidence of a marked metabolic shift associated with GLP-1 agonist treatment, with a fold enrichment of 28.4 affecting the oxidative phosphorylation pathway, representing the presence of 12 significantly downregulated genes relevant to the progression of substrates through this pathway. Other downregulated processes included those involved in the pathogenesis of a variety of cardiomyopathic states, including cardiac muscle contraction, hypertrophic cardiomyopathy, dilated cardiomyopathy, and diabetic cardiomyopathy. These pathways, along with their associated quantity of represented genes, fold enrichment, and FDR, are represented as for upregulated processes in Figure 6A. Generalization of the bioinformatic source to all available databases yielded a similar distribution of processes, including the mitochondrial respirasome, cardiac muscle contraction, and diabetic cardiomyopathy; this data is shown in Figure 6B.

**Figure 7.**
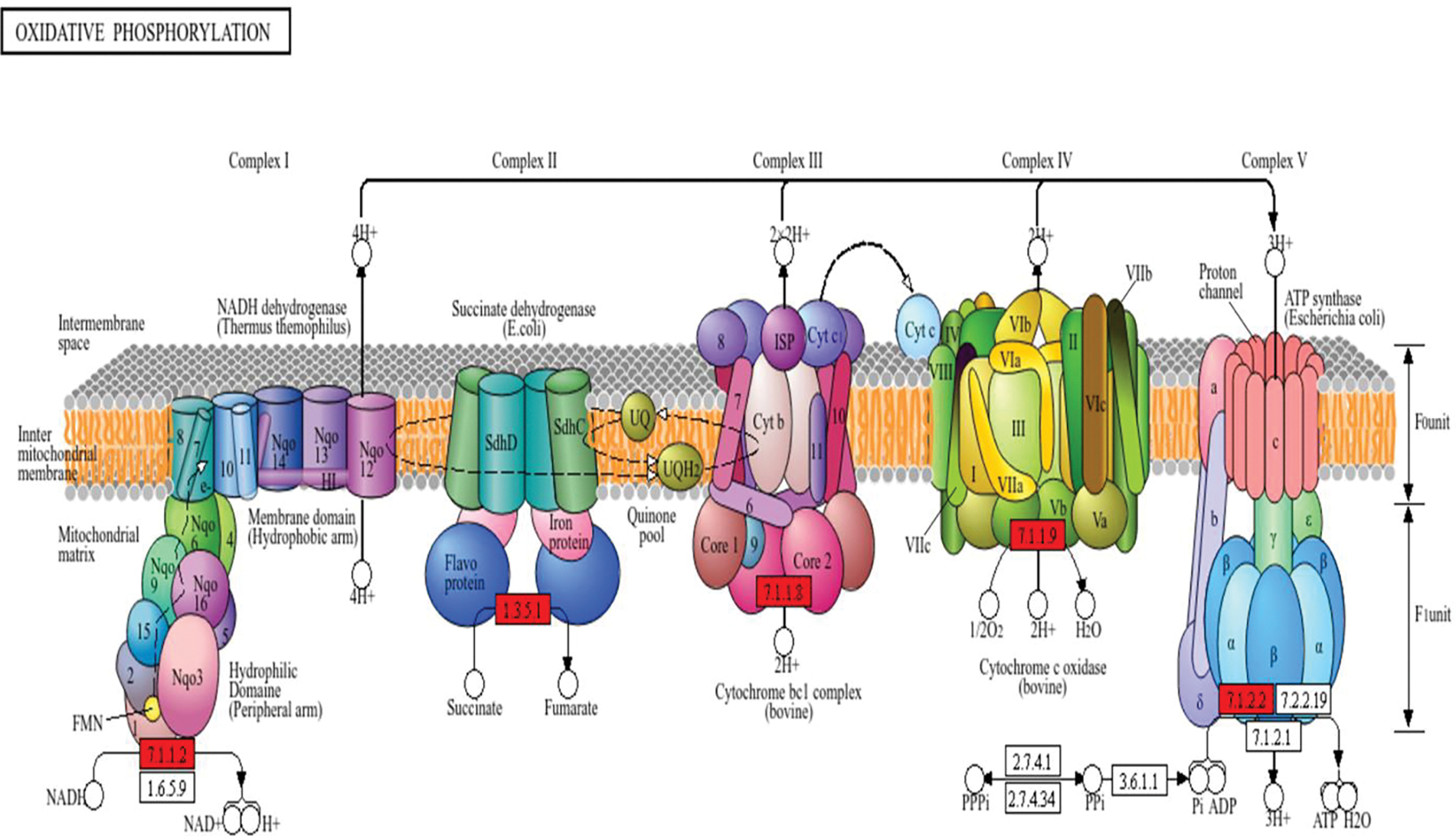
Semaglutide Downregulates Mitochondrial Respirasome Subunits. This diagram depicts the mitochondrial complexes responsible for the electron transport chain. As highlighted in red, our dataset indicated reduced expression associated with each of these complexes. 7.1.1.2: Complex I/NADH dehydrogenase. 1.3.5.1: Complex II/succinate dehydrogenase. 7.1.1.8: Complex III/cytochrome bc1 complex. 7.1.1.9: Complex IV/cytochrome c oxidase. 7.1.2.2: ATP synthase.

Given the functional detriment that would arise from the reduced myocardial capacity to translate cellular respiration into ATP, we again drew on KEGG-facilitated pathway analysis. Figure 7 displays the results for the oxidative phosphorylation pathway, illustrating reduced expression of subunits belonging to NADH dehydrogenase (Complex I), succinate dehydrogenase (Complex II), cytochrome bc1 complex (Complex III), cytochrome c oxidase (Complex IV), and ATP synthase. Additionally, see Table 2 for a listing of the minority of mitochondrial respirasome subunits individually identified within the proteomic dataset. Proteins of cardiomyopathic relevance were similarly mapped, and determined to include cardiac troponin I3 (TNI3), actin gamma 1 (ACTG1), actin alpha cardiac muscle 1 (ACTC1), tropomyosin (TPM), myosin light chain (MYL2–3), and myosin heavy chain (MYH6/7). The reduced abundance of sarcomeric structural proteins in SEM animals may indicate reduced adverse remodeling in the treated myocardium. This accords with recent trial evidence, through which semaglutide was shown reduce remodeling in patients with obesity-associated heart failure.^31^

### ADDITIONAL FINDINGS OF POTENTIAL MECHANISTIC INTEREST

Utility of bioinformatic databases notwithstanding, there are interpretive limits to their use. In acknowledgement of this, we undertook a manual survey of the significantly altered proteome for proteins of potential relevance either to ischemic heart disease pathogenesis, or to its amelioration. This revealed a multitude of targets of potential disease-modifying interest. Beyond the metabolic pathways highlighted previously, the dataset contained a variety of proteins of demonstrated importance to cardiac function and disease. The first of these is oxysterol binding protein. Although the role of this protein remains under active investigation, it is thought to be involved in lipid metabolism, and to exert a potential protective effect against atherosclerotic disease; it was upregulated over 2.5-fold (p=0.001) in our dataset.^32^ Another such protein was argininosuccinate synthetase. This protein, which was upregulated 2.2-fold (p=0.041)in semaglutide-treated animals, catalyzes the rate-limiting step of nitric oxide synthesis; not only is the vasodilatory and antithrombotic nitric oxide an essential bulwark against vascular pathobiology in general, it has also been previously shown by our lab to be central to the cardioprotective effects of semaglutide.^33,34(p11)^ Combined with the concurrent upregulation of dimethylargininase, which degrades an inhibitor of nitric oxide synthase, has been associated with augmented neovascularization, and was overexpressed 1.5-fold (p=0.002) in semaglutide-treated animals, this constitutes substantial evidence of the relevance of the nitric oxide biosynthetic pathway to an account of the cardiac effects of semaglutide.^35^

Additionally, there was overexpression in animals that received semaglutide of multiple antioxidant enzymes, including manganese superoxide dismutase (MnSOD, 1.45-fold, p=0.003). This mitochondrial enzyme has been studied extensively as a protective factor in metabolic cardiomyopathy, in the setting of which it has been shown to normalize contractile function.^36^ Moreover, MnSOD is thought by virtue of its localization to provide the principal source of protection against oxidative damage provoked by mitochondrial respiration; its activation in this tissue may therefore suggest increased or more efficient respiratory function in treated animals.^37^ Other upregulated antioxidants included perilipin 5 (1.39-fold, p=0.008)), which has been shown to optimize ATP generation efficiency by regulating the oxidation of fatty acids;^38(p5)^ and glutaredoxin (1.36-fold, p=0.024), a thioltransferase thought to provide an antioxidant-associated protective effect against coronary atherosclerosis.^39^

Finally, it is also notable, in light of the reduction in mitochondrial subunit expression seen in the downregulated dataset, that multiple respirasome proteins were also found to be upregulated, including electron transfer flavoprotein-ubiquinone oxidoreductase, which is responsible for linking fatty acid and amino acid degradation with oxidative phosphorylation and was increased 1.3-fold (p=0.023);^40^ and mitochondrial intermediate peptidase, which was increased 1.57-fold (p=0.028) and is thought to be involved in maturation of the oxidative phosphorylation complexes.^41^ Additional support for this contention is supplied by consideration of the upregulation of glycogen synthase (1.39-fold, p=0.017), the increased presence of which implies an anabolic, ATP-replete cellular state.^42^ The increased expression of mitochondrial creatine kinase (1.39-fold, p<0.001) in treated tissue provides analogous evidence, as this represents the dominant source of stored energy within the myocardium.^43^

Suggestive as these changes clearly are, they likely represent only a small minority of the full breadth of pathogenically relevant changes in this dataset, many of which may involve proteins with as-yet poorly explored functions. A comprehensive listing of the hundreds of remaining significant differences seen in the semaglutide-treated ischemic myocardium lays outside the scope of this manuscript, and raw data is available upon request to the author. We hope that, against the background of the progressive revelation of protein structure and function across the genome characteristic of recent decades, this data will serve as a durable resource for assessment of ischemic cardiomyopathic pathogenesis and protection in the years to come.^44^

## Discussion:

The 2023 assessment that GLP-1 agonist drugs were the scientific of breakthrough of the year was prescient.^45^ While certainly justified at the time by the data then available, the results and enthusiasm following use of these agents have, if anything, grown since then. Over the past year, trial evidence has continued to suggest that the true promise of GLP-1 agonists has only just begun to be mapped. Trials are underway or completed for diverse conditions, but the findings most pertinent to the treatment of cardiovascular disease arose from the SELECT trial. Not only did primary analysis reveal reduced death from cardiovascular causes and non-fatal myocardial infarction overall, but prespecified secondary analysis also showed that, among patients with atherosclerotic disease and heart failure, there were reduced composite heart failure hospitalizations and cardiovascular deaths among patients taking semaglutide.^46^ In summary, there is robust evidence that GLP-1 agonist therapy favorably impacts the trajectory of cardiovascular disease across the spectrum of severity.

Given the confinement of trial evidence to patients with the established indications for use of GLP-1 agonists, however—patients either had a BMI of 27 or greater, type 2 diabetes mellitus, or both—the literature remains incomplete. Specifically, we do not know what component of GLP-1 effects is attributable not to risk factor modulation, but rather to processes acting directly on the heart. Moreover, what is the molecular mechanism by which these processes proceed? Answering these questions would provide insights not only into the biology of GLP-1 agonism, but could also reveal novel pathways for the adjunctive optimization of CAD therapeutics.

In the first effort to furnish these answers, we used cardiac functional and coronary perfusion analysis to characterize the effects of semaglutide therapy. Using these methods, we demonstrated that semaglutide improved left ventricular ejection fraction, coronary perfusion to the ischemic territory, and reduced fibrosis and apoptosis in this region. In light of the five-week timeline and lack of metabolic comorbidities with which our study was designed, is it likely that the origin of these changes was the ischemic myocardium itself.

To substantiate this hypothesis, we subjected the ischemic myocardial tissue generated in this experiment to proteomic profiling. Proteomics represents an important subset of the high-throughput methodologies that have revolutionized the molecular investigation of disease in recent years. Using this technique, it has become possible to develop models of the interaction between diseases and potential therapies with unprecedented precision and depth. In keeping with this, the proteomic method described in this manuscript was sufficient to discover 7,322 co-expressed proteins within ischemic myocardial sections. After filtration, this total was reduced by approximately one order of magnitude, yielding 594 significantly upregulated and 90 significantly downregulated proteins in ischemic myocardial sections from animals given semaglutide.

The bioinformatic yield of these changes was striking. Accounts of the serum glycemic and body weight reductions produced by GLP-1 agonists have prompted a number of previous metabolic pathway explorations that have demonstrated activations of glycolysis, the citric acid cycle, and fatty acid degradation, converging on increased intracellular ATP.^47,30^ These effects were found in peripheral organs, such as in pancreatic β-cells and adipose tissue. Despite the existence of GLP-1 receptor expression in all four cardiac chambers in levels approximating those in the pancreas, however, the metabolic result of receptor activation at this site is largely unknown.^48^ For this reason, our evidence that semaglutide augmented expression of nearly every citric acid cycle enzyme, most glycolytic enzymes, and the rate-limiting enzymes from both of these processes, is both novel and consistent with the established effects of this agent. Taken together with prior work that established the cardioprotective effect of citric acid cycle intermediates,^49^ this evidence implies that the treated myocardium is better-equipped than that from non-treated hearts for energy generation in the ischemic environment. Ischemia has been with a metabolic shift away from β-oxidation to the use of glucose of a substrate. Thus, the enzyme expression changes produced by semaglutide likely support the metabolic sustenance of the heart in this context.^50^

Acceptance of these conclusions, of course, requires reconciliation with the finding that multiple mitochondrial respiratory complex subunits exhibited decreased expression in treated animals. If this signified reduced cellular energy generation, it would contradict the notion that the other enzymatic changes were favorable. Fortunately, there is support within our results for the notion that energy generation is improved in treated tissue. First is the appearance of multiple anabolic enzymes in the upregulated proteome. These include glycogen synthase and creatine kinase, which is the principial chemical locus of myocardial energy reserves. This aligns with the contention that this tissue exhibits an energy-replete cardiac state. If respirasome subunit reductions implied reduced ATP generation, this could then be explained as a response to the upstream surplus stored in the form of creatine kinase and glycogen. Second, as displayed in Table 2, the downregulated subunits represent a small minority of the total mass of ATP-generating mitochondrial machinery. Moreover, there were multiple components of this machinery also represented in the upregulated proteome. Taken together, these data most likely represent a more dynamic process than simple respirasome downregulation. As corroborated by upregulation of the proteasomal degradation pathway that is responsible for respirasome homeostasis, they likely indicate increased turnover of the respiratory machinery. This turnover, under control of the proteasomal system, is essential in the setting of physiologically stressful conditions such as chronic myocardial ischemia. Lacking this, dysfunctional, misfolded complexes impair ATP generation, ultimately resulting in cell death.^51^

As extensive as these metabolic changes were, and although they possess concordance with the functional and vascular changes previously demonstrated in our lab, they must be acknowledged to be preliminary and limited in their failure to display the full profile of ischemic myocardial GLP-1 agonism. This would require concurrent metabolomics, as this would display the quantity of intermediates correlating with the enzymatic state of assayed cells. Similarly, it is now possible to perform spatially-differentiated multi-omic tissue profiling, such that the content of DNA, production of transcripts, and expression of proteins thus discovered can be differentiated according to precise cellular, or even sub-cellular, origin.^52^ Finally, it is important to mention that the filtration parameters used in our experiment result in inclusion only of a small minority of the entire ischemic myocardial proteome within our analytic purview, which would be meaningfully altered if different parameters were used. In particular, our use of an expressive fold change threshold of 1.1 resulted in the inclusion of a relatively larger breadth of significantly altered proteins. This may have come at the expense, however, of specific emphasis on myocardial proteins with more marked expressive changes. This, considered in conjunction with the fact that our sample size was relatively small, selected as it was according to power equations previously performed by our lab^18^, limits the generalizability of these results. In this context, our data must be viewed as exploratory and hypotheses-generating in nature, and presented for the purpose of providing suggestions for future, more targeted investigations of the myocardial biology of GLP-1 agonism. Accordingly, we hope that our results will serve as a basis for comparison for subsequent efforts within the field to expand on and validate these findings. Until this time, this experiment has provided the most through molecular exegesis of the clinical efficacy of semaglutide for patients with ischemic cardiomyopathy currently available.

## Conclusions:

In summary, we subjected ischemic porcine myocardial tissue to data-independent acquisition proteomics following generation of a data-dependent spectral library. This permitted a comprehensive and highly sensitive molecular comparison between hearts exposed to oral semaglutide therapy, and those that underwent ischemia induction alone. Analysis of this comparison revealed marked metabolic modulation in the treated tissue. This conforms to the adaptive program known to be instituted in the myocardium following subjection to ischemic insults. Correspondingly, this implies that the cardiac benefits of GLP-1 agonism may arise in part from this metabolic support. There were also downregulations observed in subunits of oxidative phosphorylation enzymes, but these were counterbalanced by concurrent significant upregulations in other subunits. The rate-limiting enzyme of glycogen synthesis and the principal enzyme responsible for myocardial energy reserve maintenance were also upregulated, as was the proteasomal process by which the respirasome is recycled to maintain its functionality. Other downregulations involved the sarcomeric apparatus. This may account for the newly characterized capacity of semaglutide to mitigate pathologic remodeling in patients with heart failure.

## Figures and Tables

**Table 1.** Treated Ischemic Myocardial Metabolic Shifts.

Enrichment FDR	Number of Genes	Fold Enrichment	Pathway
2.8E-07	7	2	2-Oxocarboxylic Acid Metabolism
1.4E-10	11	19.8	Glyoxylate and Dicarboxylate Metabolism
2.5E-34	37	18.2	Carbon Metabolism
2.2E-18	20	18	Glycolysis / Gluconeogenesis
3.1E-08	9	16.8	TCA Cycle
2.6E-19	22	16.7	Biosynthesis of Amino Acids
14.1E-08	9	16.2	Propanoate Metabolism
2.7E-09	11	14.9	Pyruvate Metabolism
9.9E-07	9	11.3	Proteasome
1.7E-05	9	9.6	Aminoacyl-tRNA biosynthesis
5.9E-06	9	9.2	Legionellosis
8.6E-07	12	7.5	mRNA Surveillance Pathway
7.2E-08	15	6.9	AMPK Signaling Pathway

Pathway enrichments within semaglutide-treated ischemic myocardial tissue. FDR: false discovery rate. TCA: tricarboxylic acid cycle. tRNA: transfer ribonucleic acid. mRNA: messenger RNA. AMPK: adenosine monophosphate-activated protein kinase.

**Table 2.** Mitochondrial Respirasome Subunit Reductions.

Complex I	Complex II	Complex III	Complex IV	Complex V
ND1, ND2, ND3, ND4, **ND5**, ND6, Ndufs1, Ndufs2, Ndufs3, **Ndufs4**, Ndufs5, Ndufs6, **Ndufs7**, Ndufs8, Ndufv1, Ndufv2, Ndufv3, Ndufa1, Ndufa2, **Ndufa3**, **Ndufa4**, Ndufa5, Ndufa6, Ndufa7, Ndufa8, Ndufa9, Ndufa10, Ndufab1, Ndufa11, Ndufa12, **Ndufa13**, Ndufb1, Ndufb2, Ndufb3, **Ndufb4**, Ndufb5, Ndufb6, Ndufb7, Ndufb8, Ndufb9, Ndufb10, Ndufb1, Ndufc1, Ndufc2	SDHA, **SDHB**, SDHC, SDHD	**ISP**, Cytb, Cyt1, COR1, QCR2, QCR6, QCR7, QCR9, QCR9, QCR10	COX1, COX2, COX3, COX4, COX5A, COX 5B, **COX6A**, **COX6B**, COX6C, COX7A, COX7B, COX7C, COX8, COX10, COX11, COX15, COX17, CYC	Alpha, Beta, Gamma, Delta, Epsilon, OSCP, a, b, c, d, e, f, g **f6/h**, j, k, 8

In this table, individual subunit reductions are recorded. Reductions are rendered in bold, while the remainder of subunits found in eukaryotes appear in plain text.

## Data Availability

All data not contained either in this manuscript or elsewhere in the published literature (e.g., microsphere quantification, raw pressure-volume loop tracings, raw proteomic values, etc.) but that are germane to the full evaluation of results are fully available upon request made to the corresponding author.
